# Impact of Genomic Deletion RD16 on the Expression of the *Mycobacterium bovis* BCG Moreau VapBC47 Toxin-Antitoxin System

**DOI:** 10.3390/cimb45080412

**Published:** 2023-08-07

**Authors:** Talita Duarte Pagani, Paloma Rezende Corrêa, Cristiane Lima, Leonardo Henrique Ferreira Gomes, Marcos Gustavo Araujo Schwarz, Teca Calcagno Galvão, Wim Maurits Degrave, Napoleão Fonseca Valadares, Leila Mendonça-Lima

**Affiliations:** 1 Laboratório de Genômica Funcional e Bioinformática, Instituto Oswaldo Cruz, Fiocruz, Rio de Janeiro 21040-360, RJ, Brazil; tdpagani@yahoo.com (T.D.P.); pah.rez.correa@gmail.com (P.R.C.); crikatheryn@gmail.com (C.L.); leonardo.henrique@fiocruz.br (L.H.F.G.); schwarz@ioc.fiocruz.br (M.G.A.S.); wim.degrave@fiocruz.br (W.M.D.); 2 Laboratório de Bacteriologia, Centro de Referência Prof. Hélio Fraga, Escola Nacional de Saúde Pública Sergio Arouca, Fiocruz, Rio de Janeiro 21041-210, RJ, Brazil; teca@ioc.fiocruz.br; 3 Departamento de Biologia Celular, Universidade de Brasília, Brasília 70904-970, DF, Brazil; napoleaofv@gmail.com

**Keywords:** *Mycobacterium bovis* BCG Moreau, *Mycobacterium tuberculosis*, toxin–antitoxin system, VapBC47, BCG vaccine

## Abstract

*Mycobacterium bovis* BCG is the only vaccine against tuberculosis. The variable forms of cultivation throughout the years, before seed-lots were developed, allowed in vitro evolution of the original strain, generating a family of vaccines with different phenotypic and genotypic characteristics. Molecular studies revealed regions of difference (RDs) in the genomes of the various BCG strains. This work aims to characterize the gene pair *rv3407-rv3408* (*vapB47*-*vapC47*), coding for a toxin–antitoxin system of the VapBC family, and to evaluate possible transcriptional effects due to the adjacent BCG Moreau-specific genomic deletion RD16. We show that these genes are co-transcribed in BCG strains Moreau and Pasteur, and that the inactivation of an upstream transcriptional repressor (Rv3405c) due to RD16 has a polar effect, leading to increased *vapBC47* expression. Furthermore, we detect VapB47 DNA binding in vitro, dependent on a 5′ *vapB47* sequence that contributes to a palindrome, spanning the promoter and coding region. Our data shed light on the regulation of VapBC systems and on the impact of the BCG Moreau RD16 deletion in the expression of adjacent genes, contributing to a better understanding of BCG Moreau physiology.

## 1. Introduction

Tuberculosis (TB) is a widespread infectious disease estimated to affect one third of the global population. It ranked first among infectious diseases causing deaths worldwide, only now surpassed by COVID-19. Despite great efforts, the TB death toll is still exceedingly high, with an estimated 1.3 million deaths among HIV-negative people and an additional 214,000 among HIV-positive people in 2020. The decline in TB incidence achieved in previous years has almost stopped [1]. Although most diagnosed TB patients can be cured, the recommended treatment takes 6 months and employs four drugs [1]. Multidrug-resistant TB (MDR-TB) is another concern. With 157,903 new cases in 2020 (a significant 22% reduction compared to 2019), the treatment of MDR-TB is more expensive and can take twice as long [1].

*Mycobacterium tuberculosis* (*Mtb*) resorts to several molecular mechanisms to achieve its well-known resilience, among these being a wide array of toxin–antitoxin systems. The *Mtb* genome encodes TA pairs belonging to several well-known type II TA system families, such as VapBC, MazEF, ParDE, RelBE and HigBA. VapBC is not only the most abundant within the *Mtb* genome but the most studied TA family for this pathogen [2]. Nonetheless, other TA family pairs also impact *Mtb* biology, such as MazEF, which can aid in the infection process by degrading host RNA, since MazF proteins are secreted [3]. The genome of *Mtb* H37Rv carries at least 88 toxin–antitoxin (TA) loci, 47 of which are VapBC type II TA systems, with roles in adaptation to stress and metabolic reprogramming [3]. In VapBC TA systems, the genes coding for the antitoxin VapB and the toxin VapC form an operon, thus being co-transcribed from a mutual promoter; the antitoxin precedes toxin expression, ensuring effective neutralizing activity [4]. VapC toxins usually fold into a PIN (PilT N-terminus) domain associated to ribonuclease activities [5,6,7], while the antitoxins present a C-terminal region of variable length that binds to their cognate toxin, inhibiting its action. Additionally, the N-terminal region of the antitoxin can bind to a DNA sequence in its own promoter, regulating the expression of both toxin and antitoxin [8]. Most of the time, VapCs are inhibited by their cognate VapBs, but the antitoxins might be subject to the action of proteases, leaving the toxin free to exert its ribonuclease activity [9]. This in turn is mediated by the recognition of a site in the target RNA, such as a stable hairpin, as is the case of the posttranscriptional control of operons involved in sugar metabolism in *M. smegmatis* [10].

VapBC systems are associated with persistence, a transient multidrug tolerance state marked by a very low metabolic rate that arises from stochastic changes in normal cells [8,11,12]. It has been suggested that the ribonuclease activity of VapC has an impact on the differential decay of RNA in the cell and the reduction in the expression of several proteins, contributing to the persistence phenotype [12]. Persistence is also thought to be related to the development of multidrug resistance, since some persisters might be able to survive in the host during antibiotic treatment [9,11,13].

These characteristics make VapBC TA pairs interesting targets in the mycobacteria research field. While most bacteria have only one VapBC pair in the genome, their diversity in *Mtb* and other mycobacteria indicates elaborate molecular mechanisms to deal with diverse adverse conditions. This may contribute to the evolutionary success of several mycobacteria as pathogens, enabling them to lead a life cycle with dramatically different endpoints depending on the conditions encountered throughout the infection process. This could impact antigen expression levels and/or metabolic rates, thus aiding cells to enter into a less immunogenic dormancy state [14]. Some of *Mtb* VapBC systems act on degrading rRNA, such as VapC20 and VapC26 acting on the 23S rRNA [5,15], while others act on tRNA, such as VapC2 and VapC21 acting on tRNA^fMet^ [14] and VapC4 on tRNA^Cys^ [16]. The overall effect of toxin action leads to translation inhibition, either by ribosome inactivation or reduction of the tRNA pool, resulting in a slower translation rate.

Besides *Mtb*, another *Mycobacterium* of great relevance is *M. bovis* BCG, the attenuated strain obtained by Calmette and Guérin from a virulent *M. bovis* clinical isolate. The original vaccine strain was distributed worldwide in the 1920s and maintained under variable culture conditions, leading to genomically heterogeneous BCG daughter strains. Among these is BCG Moreau, the strain used for Tuberculosis vaccination in Brazil since 1928 (reviewed in [17]). A feature unique to BCG Moreau is RD16, a 7608 bp genomic deletion resulting in the loss of genes *rv3401*-*rv3404c* and truncation and fusion of the remaining *rv3400* and *rv3405c* sequences, directly adjacent to *rv3406*, *rv3407* and *rv3408* relative to the *Mtb* H37Rv genome annotation [18,19].

In *Mtb* and in *M. bovis* BCG Pasteur (a reference BCG strain), *rv3405c* encodes a TetR transcription regulator [20] while *rv3407* and *rv3408* code for the VapBC47 TA system, as annotated and observed in the online tool Mycobrowser [21]. Rv3405c was shown to repress *rv3406* transcription in BCG Pasteur, and its truncation in BCG Moreau results in constitutive Rv3406 (BCGM3440) expression [20]. The proximity of *rv3407* and *rv3408* to the strong *rv3406* promoter raises the hypothesis that regulation of this toxin–antitoxin system, corresponding to ORFs *BCGM3441* and *BCGM3442*, could be affected in BCG Moreau.

Regulation of VapBC systems has been studied in *Mtb* and *M. smegmatis* but remains poorly explored in *M. bovis* BCG [22]. Its role in bacterial persistence and proximity to RD16 in BCG Moreau makes VapBC47 an appealing target for the study of the molecular mechanisms of regulation of VapBC expression. Interestingly, although the *Mtb* antitoxin VapB47 (Rv3407) has been shown to improve protection against tuberculosis when tested as a DNA vaccine [23], to date there is no report concerning its TA functionality.

Here we show that *vapB47* and *vapC47* are co-transcribed in BCG strains Moreau and Pasteur, with higher transcriptional levels in BCG Moreau possibly due to a polar effect resulting from *rv3405c* inactivation, a direct consequence of RD16 on the expression of this toxin–antitoxin system. Additionally, we demonstrate that recombinant VapB47 binds the *rv3406*-*rv3407* intergenic region (IR*06*-*07*) in vitro, and the interaction depends on the presence of the *vapB47* 5´ coding region. Taken together, these results highlight important mechanisms involved in the regulation of the VapBC47 toxin–antitoxin system in *M. bovis* BCG.

## 2. Materials and Methods

### 2.1. Bacterial Strains and Culture Conditions

*Escherichia coli* Top10 and BL21 (DE3) strains were used as hosts for cloning and recombinant protein expression, respectively. *E. coli* was grown in Luria-Bertani (LB) medium (Merck, Darmstadt, Germany) at 37 °C and kanamycin (50 µg/mL) was used for plasmid selection and maintenance. BCG strains Moreau and Pasteur (obtained as described in [24]) were grown from frozen stocks kept at −80 °C and cultivated as surface pellicles in Sauton medium at 37 °C without agitation for approximately fifteen days or in Middlebrook 7H9 broth (Difco, MD, USA) containing 0.2% (*v*/*v*) glycerol, 0.05% (*v*/*v*) Tween 80 and 10% ADC (5% [*w*/*v*] albumin, 0.003% [*w*/*v*] catalase, 2% [*w*/*v*] dextrose) with agitation at 37 °C.

### 2.2. RNA Extraction and Reverse Transcription-Polymerase Chain Reaction (RT-PCR)

Total RNA from *M. bovis* BCG Moreau and Pasteur cultured for 15 days in Sauton medium was extracted using TRIzol (Thermo Fisher Scientific, Waltham, MA, USA) and RiboPureTM Bacteria kit (Thermo Fisher Scientific, Waltham, MA, USA), according to the manufacturer’s protocol. Briefly, cells were lysed with zirconium beads using bead-beater equipment (Biospec Products Inc., Bartlesville, OK, USA). After chloroform extraction, the supernatant was passed through the column and RNA was further eluted in RNAse-free water. DNA contaminants were removed by Turbo-Dnase (Thermo Fisher Scientific, Waltham, MA, USA) digestion, according to the manufacturer’s instructions. cDNA was synthesized from 600 ng of total RNA using random primers and the SuperScript III First-Strand Synthesis SuperMix for RT-qPCR kit.

Assays were performed to detect co-transcription of different targets, using distinct pairs of primers: for *rv3406*-*rv3407* (RT-06F: ACGGCGAATCACCATGCCCG; RT-07R: GACGACGAGCCGGAATCAGG) and *rv3407*-*rv3408* (RT-07F: GCGTGCTACCGTTGGGCTTG; RT-08R: TGAGGCGGTGACGAAGCCGA). PCR cycling conditions were: 95 °C/5 min (1 cycle); 95 °C/30 s, 60 °C/ 30 s, 72 °C/30 s (35 cycles); 72 °C/7 min (1 cycle). RT-PCR experiments were performed in triplicates.

### 2.3. Quantitative RT-PCR (RT-qPCR)

RT-qPCRs for quantification of *rv3407* and *rv3408* transcripts in BCG Moreau and Pasteur cultured in Sauton medium for 15 days at 37 °C were performed. For this assay, 2 ng of cDNA were used in a final reaction volume of 25 μL, containing 12.5 μL of Power Green PCR Master Mix and 100 µM (each) of forward and reverse primers (for *rv3407*: RT-07F and RT-07R; for *rv3408*: RT-08F [CTCGACGGGCTCGACATCCT] and RT-08R). *sigA* transcription (with primers SigA-F: CTCGACGCTGAACCAGACT and SigA-R: AGGTCTTCGTGGTCTTCGC) was used for internal normalization. PCR reactions were conducted in a 7500 Real Time PCR System (Applied Biosystem, Waltham, MA, USA) in 96 well plates with the following conditions: 95 °C/10 min (1 cycle); 95 °C/30 s, 60 °C/30 s, 72 °C/30 s (40 cycles). A dissociation curve was performed after each assay to check the quality of each primer. Gene expression levels were calculated by relative quantitation using the comparative Ct method (ΔΔCt), as previously described [25]. mRNA relative levels were normalized against *sigA* mRNA [26,27] and qPCR experiments were performed in triplicates.

### 2.4. RT-qPCR Statistical Analysis

Differences obtained from comparative RT-qPCR of *M. bovis* BCG strains Moreau and Pasteur were analyzed with the Student’s *t*-test to determine significant differences among group means. First, data were submitted to the Kolmogorov–Smirnov test and the Shapiro–Wilk test, pointing to a normal distribution. Statistical analysis was carried out using the data obtained from different sets of independent biological samples. A *p*-value ≤ 0.05 was considered as statistically significant.

### 2.5. Expression and Purification of Soluble Recombinant Rv3407

For cloning, expression and purification of recombinant Rv3407 (rRv3407) containing a C-terminal His-tag, the *rv3407* coding region was amplified from BCG Moreau genomic DNA using primers pET-07F (GGCGCCATGGGTGCTACCGTTGGGCTT) and pET-07R (CCGGCTCGAGCTGCTCGTCGCGCATTTC) and the purified 314 bp PCR product was digested with *Nco*I and *Xho*I and ligated into pET28a vector DNA, digested with the same enzymes, using T4 DNA ligase ( Thermo Fisher Scientific, Waltham, MA, USA). A five-time diluted ligation reaction was used to transform *E. coli* Top10 electrocompetent cells, and colonies, after antibiotic selection, were tested for the presence of inserts by colony PCR. Integrity of the insert present in purified plasmid DNA from a positive clone was also confirmed by DNA sequencing. Following transformation into *E*. *coli* BL21 (DE3), an isolated colony harboring pET28a-*rv3407* was grown in 10 mL LB containing 0.5% [*w*/*v*] glucose and 50 µg/mL kanamycin at 37 °C until OD_600nm_ = 0.7. Cells were recovered by centrifugation and transferred to 500 mL Overnight Express™ Instant TB Medium (Novagen, Darmstadt, Germany) containing 50 µg/mL kanamycin. The culture was incubated at 37 °C for 16 h with agitation. Bacterial cells were harvested, resuspended in 30 mL of lysis buffer (50 mM Tris-Cl pH 7.5, 300 mM NaCl, 0.5% [*v*/*v*] Triton X-100, 10 mM β-mercaptoethanol, 1 mg/mL lysozyme, 1 µg/mL DNase, 5 mM imidazole, 1 capsule of Roche EDTA-free protease inhibitor), and cells were disrupted by sonication. The lysate was clarified at 12,000× *g* for 20 min at 4 °C and the soluble fraction was loaded into a Ni^2+^ immobilized-metal affinity chromatography (IMAC) column, which was washed in buffer A containing 20 mM Tris-CL pH 7.5 and 100 mM KCl. The elution of rRv3407 was achieved by applying a linear imidazole gradient (20 mM–500 mM) in buffer A. After evaluation by electrophoresis in reductive/SDS-denaturant polyacrylamige gel (SDS-PAGE), fractions containing purified rRv3407 were pooled and the protein concentrated using an Amicon Ultra–4 ultracel 30 kDa (Millipore, Darmstadt, Germany). Buffer was exchanged four times using an Amicon Ultra–4 ultracel 5 kDa (Millipore, Darmstadt, Germany) to 10 mM Tris-Cl, pH 7.5, 100 mM NaCl, 0.05% (*v*/*v*) Tween 20.

### 2.6. Native PAGE and Electrophoretic Mobility Shift Assays (EMSA)

For EMSA assays, two biotinylated DNA fragments were obtained by amplification using PCR Master Mix 2 X (Thermo Fisher Scientific, Waltham, MA, USA). The first, using primers 07promFOR biotinylated (TCGACGTGTACGGGCAGGCT) and 07promREV1 (GGTGCTACATAATTGCGTTG) yielded a fragment of 96 bp comprising the end of the *rv3406* gene (62 bp) and the intergenic region between *rv3406* and *rv3407* (IR*06*-*07*; 34 bp). The second, using primers 07promFOR biotinylated and 07promREV2 (TCCACAAGCCCAACGGTAGC) yielded a fragment of 122 bp, containing the same region described above plus the first 26 bp of *rv3407*. Cycling conditions for PCR were: 95 °C/4 min (1 cycle); 95 °C/30 s, 53 °C/30 s, 72 °C/30 s (35 cycles); 72 °C/4 min (1 cycle). The PCR products were used as probes in EMSA reactions. Briefly, the reactions were set up at 4 °C for 1 h in 20 mM Tris-Cl pH 7.5, 100 mM KCl, 2.5% (*v*/*v*) glycerol, 0.05 µg/µL poly dI-dC, 0.5 µM of the shorter or longer biotin-labelled DNA fragments, and purified rRv3407, in different concentrations (0; 0.4; 1.2; 3.5; 10.6 µM). Pre-electrophoresis of 5% polyacrylamide native gel was performed in 0.5 X TBE buffer at 4 °C for 30 min at constant 100 V. Reactions were resolved by native 5% PAGE, run at 85 V at 4 °C in TBE 0.5 X, and the results were visualized with the LightShift^®^ Chemiluminescent EMSA kit (Thermo Scientific, Waltham, MA, USA) [28].

## 3. Results

### 3.1. vapB47 and vapC47 Are Co-Transcribed in BCG MOREAU and Pasteur

**Figure 1:**
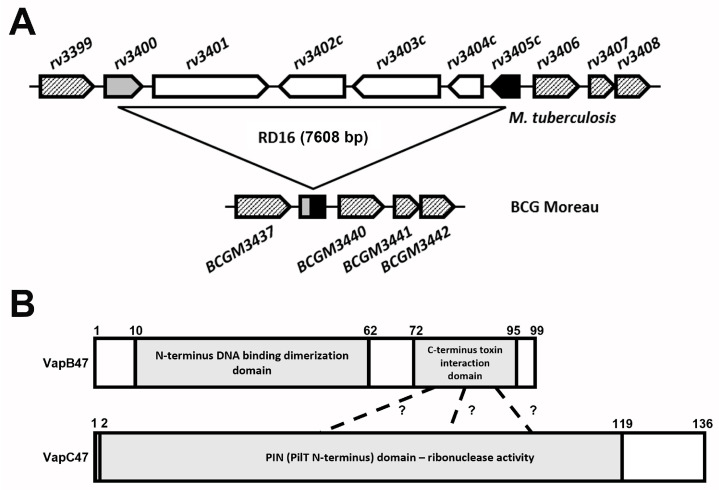
Schematic representation of RD16 and VapBC47. (**A**) *Mtb* (upper panel) and BCG Moreau (lower panel) genomic regions showing RD16 and adjacent genes. In BCG Moreau, RD16 removes genes *rv3401*, *rv3402c*, *rv3403c* and *rv3404c* (white arrows) and results in the fusion of *rv3400* (grey arrow) and *rv3405c* (black arrow). Genes are not drawn to scale. (**B**) VapB47 (antitoxin) and VapC47 (toxin) PFAM domains, highlighting VapB47 N-terminal DNA binding dimerization domain and its C-terminal toxin interaction domain (indicated by dashed lines, where the precise interaction residues are still unknown for this TA pair), and the PIN domain of VapC47 responsible for its ribonuclease activity. Both panels were created using presentation software and *Mtb* and BCG Moreau genomic data (for (**A**), Genbank accession numbers were NC_000962 and AM412059, respectively) and BCG Moreau VapB47 and VapC47 protein sequences and PFAM analysis (for (**B**)).

RD16 is a BCG Moreau-specific genomic deletion that led to the loss of several genes and truncation of *rv3405c*, encoding a TetR family transcriptional repressor. Immediately downstream is *rv3406*, a gene known to be repressed by Rv3405c, followed by *rv3407* and *rv3408*, coding for the VapBC47 TA pair (Figure 1A).

Figure 1B summarizes the PFAM domain structure predicted for the VapBC47 TA pair [29]. Searching within its primary structure, VapB47, the antitoxin, has a N-terminal DNA binding dimerization domain (residues 10–62) belonging to the Antitoxin Phd_YefM type II toxin–antitoxin system family, and a C-terminal toxin interaction domain (residues 72–95). Additionally, VapC47, the toxin, has a PIN-domain with ribonuclease activity. By domain homology, this region is predicted to interact with VapB47 C-terminus, leading to the inactivation of its enzymatic activity and enabling the VapB47 N-terminal DNA binding domain to dimerize and interact with its own promoter, auto-regulating its own expression (Figure 1B). This hypothesis is corroborated by similar events occurring with prophage 1 homologues, Phd (antitoxin) and Doc (toxin) proteins [30,31].

**Figure 2:**
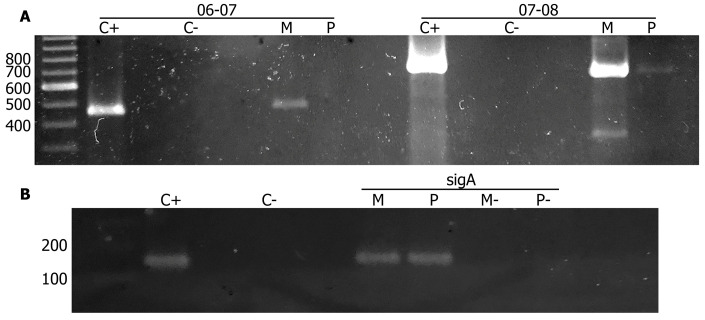
Transcription analysis of *rv3405c* downstream genes in BCG Moreau and BCG Pasteur. (**A**) RT-PCR with primers detecting *rv3406*-*rv3407* and *rv3407*-*rv3408* mRNA shows co-transcription of *rv3406*-*rv3407* (06-07) only in BCG Moreau, while the TA pair encoded by *rv3407-rv3408* (07-08) is co-transcribed in both BCG strains, as expected. (**B**) *sigA* transcription was used as an internal normalization control. Amplification of BCG Moreau genomic DNA used as positive PCR control (C+) while absence of nucleic acids was used as negative PCR control (C-). PCR directly on RNA samples not-treated with reverse transcriptase was used in Moreau (M-) and Pasteur (-) samples, confirming the absence of DNA contamination. DNA size standards indicated to the left, in bp.

Sequence analysis of the *vapBC47* coding region suggests that these genes are organized as an operon, possibly displaying translational coupling due to overlapping stop and start codons. To confirm this polycistronic organization, we performed RT-PCR on RNA samples obtained from both BCG Moreau and Pasteur strains using primers listed in Section 2.2. Our results confirm co-transcription of the *rv3407*-*rv3408* gene pair in both BCG strains (Figure 2A, right lanes), but with differences in mRNA levels. We also detect co-transcription of *rv3406*-*rv3407* in BCG Moreau (Figure 2A, left lanes), reinforcing the hypothesis of a polar effect due to RD16 in this strain. Detection of *sigA* mRNA was used as control (Figure 2B).

### 3.2. Deletion of RD16 Impacts Transcription of rv3407-rv3408 in BCG Moreau

**Figure 3:**
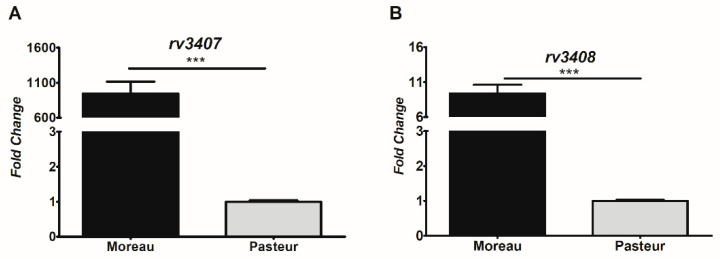
Quantitative evaluation of *vapBC47* transcripts in BCG Moreau and Pasteur. RT-qPCR analysis with primers detecting *rv3407* (**A**) and *rv3408* (**B**) mRNA corroborates the polar effect due to RD16 loss in the expression of the TA pair in BCG Moreau when compared to Pasteur. *sigA* transcription was used as an internal normalization control. *p*-value: *** < 0.001.

To assess whether the loss in regulation due to RD16 could have a polar effect on the transcription levels of the adjacent *vapBC47* (*rv3407*-*rv3408*) TA-pair, we performed quantitative real-time PCR (RT-qPCR) (Figure 3) on RNA extracted from BCG strains Moreau and Pasteur. Higher levels of *rv3407* and *rv3408* transcripts were detected in BCG Moreau compared to BCG Pasteur.

### 3.3. Rv3407 Binds to the Region Upstream of rv3407

**Figure 4:**
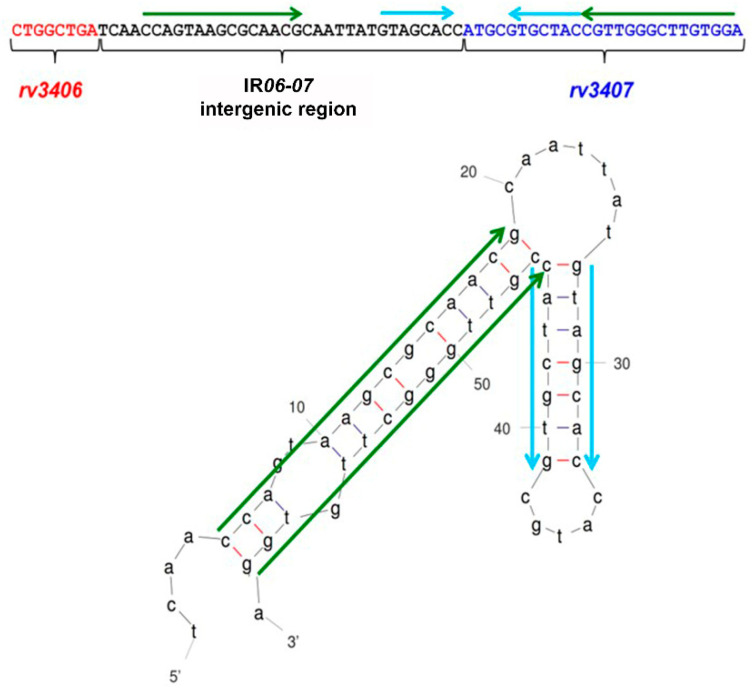
Identification of inverted repeats in the intergenic region between *rv3406-rv3407* (IR*06-07)* and the 5′ coding region of *rv3407*. The upper panel shows the DNA sequence corresponding to the end of *rv3406* (red), the IR*06-07* intergenic region (black) and the beginning of *rv3407* coding region (blue). Green and cyan arrows indicate distinct inverted repeats present in this region, represented in the lower panel (Mfold).

Inverted repeats are often present in the regulatory regions of TA genes, such as described for *Mtb* [32]. Analysis of the *vapB47* upstream region with the MFold program [33] identifies two inverted repeats (Figure 4), both involving the intergenic region between *rv3406* and *rv3407* (IR*06-07*) and the 5´ end of the *rv3407* coding region, similar to what is described for FitAB from *Neisseria gonorrhoeae* (PDB 2H1O) [34,35].

**Figure 5:**
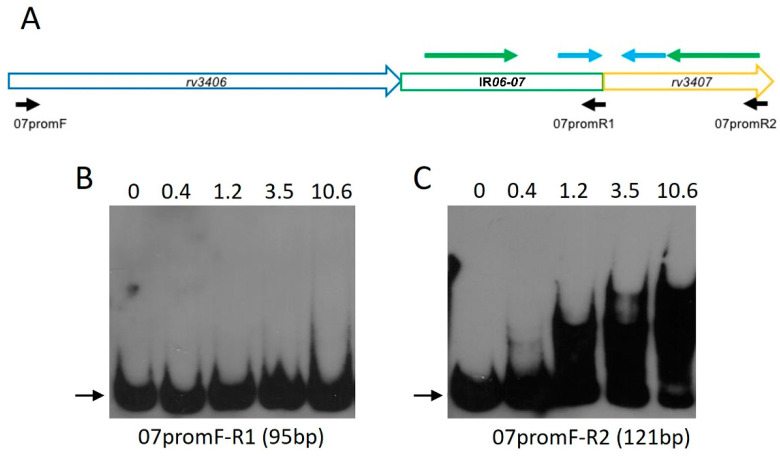
rVapB47 requires the *rv3407* 5′ region for DNA binding. The diagram (**A**) details the region corresponding to the final 62 bp of *rv3406* (blue open arrow), the 34 bp intergenic region (green box) and the first 26 bp of *rv3407* (yellow open arrow). The position of the two identified inverted repeats is indicated by the green and cyan arrows, as well as the position of the oligonucleotides used to amplify the DNA fragments for the EMSA assays (black arrows). EMSA assays to verify binding of purified rVapB47 (increasing protein amounts indicated above the lanes, in µM) to 0.5 µM of the shorter (96 bp, primers 07promF/07promR1) (**B**) or longer (122 bp, primers 07promF/07promR2) (**C**) biotin-labelled DNA fragments, respectively.

To confirm the binding of BCG VapB47 to this region, electrophoretic mobility shift assays (EMSA) were performed with purified recombinant VapB47 and biotin-labeled DNA fragments 07promF-R1 (96 bp) and 07promF-R2 (122 bp). Both fragments include the last 62 bp of *rv3406* and the IR*06*-*07* region, but 07promF-R2 also contains the initial 26 bp of the *rv3407* coding region (Figure 5A). rVapB47 does not bind to the shorter fragment (Figure 5B) but is able to bind to the fragment containing the first 26 pb of *rv3407* (Figure 5C).

## 4. Discussion

Due to their potential role as targets for antibacterial therapy, toxin–antitoxin systems are being extensively studied in *Mtb* [36,37,38,39]. The release of the BCG Moreau genome [19] allowed the identification of 47 proteins with PIN domains, the same number of the VapBC TA system described for *Mtb* H37Rv and BCG Pasteur. In particular, the location of the VapBC47 TA pair (encoded by *rv3407*-*rv3408* in *Mtb*), adjacent to the BCG Moreau-specific RD16 genomic deletion, is noteworthy. We previously described the impact of RD16 on *rv3406* expression due to inactivation of its repressor protein, Rv3405c, in BCG Moreau. Here we investigated a possible polar effect of this loss of regulation on the downstream orthologues, *rv3407*-*rv3408*, encoding this TA pair.

Our results show that the RD16 deletion leads to co-transcription of *rv3406* and *rv3407* in BCG Moreau, due to the loss of repression by Rv3405c and consequent constitutive *rv3406* expression. This directly affects the transcription of *vapB47* (*rv3407*) and *vapC47* (*rv3408*), increasing their transcription levels in comparison to the reference strain, BCG Pasteur (Figure 3), although further work is needed in order to evaluate the impact on protein levels. Semi-quantitative RT-PCR assays show that, as expected, *vapB47*-*vapC47* are co-transcribed in BCG Moreau and Pasteur, thus characterizing an operon with overlapping open reading frames and providing evidence for the existence of a common promoter upstream of *rv3407*-*rv3408*. Analysis of the intergenic region between *rv3406* and *rv3407* (IR*06*-*07*), including the first 26 nucleotides of the *rv3407* coding region, shows the presence of palindromic sequences and motifs characteristic of regulator sequences within promoter regions (Figure 4) [33,40]. Therefore, we tested this prediction by EMSA assays using recombinant VapB47, confirming that it can bind to the region upstream of its own coding gene, and that this depends on the presence of the first 26 bp of its coding region (Figure 5).

Previous studies indicate the importance of this TA pair for the *Mtb* infection process. Analyzing a RD1-deleted H37Rv strain, Mostowy et al. [41] identified genes with altered expression profiles when compared to wild-type H37Rv, and among the up-regulated ones they identified *rv3407* and *rv3408*. RD1 is a region deleted in all BCG strains when compared to virulent members of the *Mtb* complex, and it is thought to be one of the evolutionary steps towards attaining the avirulent phenotype of the BCG vaccine family. Additionally, Rv3407 is a known *Mtb* latency-related antigen and the focus of several studies as an interesting target for a DNA vaccine. It was first tested in a prime-boost protocol together with BCG, enhancing efficacy of vaccination [23]. Liang et al. [42] showed that, when used alone, the *rv3407* DNA vaccine had a protective effect, but it also showed therapeutic properties, lowering the bacillary burden within infected mice lungs. Therefore, our findings showing higher *vapBC47* expression in BCG Moreau when compared to Pasteur could be related to its specific vaccine features. In this scenario, the higher antitoxin production could have a natural Rv3407 booster effect, as observed for *Mtb*.

It was proposed for VapBC22 in *Mtb* that an imbalance in the relative levels of toxin (VapC22) and antitoxin (VapB22) results in reduced expression of various proteins, such as Esx-I, Esx-V, KasA, KasB and AcpM, as the antitoxin can regulate expression of these genes [43]. These virulence-associated proteins are known to modulate the host immune response, allowing an enhancement in intracellular *Mtb* replication. Repression of these genes is therefore related to a faster bacterial clearance by the innate immune response, resulting in reduced immunopathology [43]. If a similar mechanism occurs with VapBC47, RD16 deletion could have an impact on virulence of BCG Moreau as compared to other BCGs. A polar effect due to *rv3406* constitutive expression alters *rv3407*-*rv3408* levels, and an imbalance in these proteins’ ratio could impact the production of other virulence-associated genes that remain in the BCG genome. However, this hypothesis remains to be verified.

A study on another *Mtb* TA system, VapBC46, shows that besides producing the toxin, distinct VapC46 expression levels can lead to different pathways within the bacteria life cycle. Under a toxin threshold, VapC46 modulates the expression of several genes, while maintaining metabolic active cells; above this level, cells are directed to a dormancy state [44]. If similar events occur for VapBC47, BCG Moreau and Pasteur may take different pathways throughout the infection process resulting from the immunization event. The identification of specific RNA target(s) for VapC47 will provide additional information, helping to unravel the molecular mechanisms associated with this TA pair in regulating cell destiny, and on the possible adverse conditions that trigger its response. For example, VapC4 acts on tRNA^Cys^, reducing its level in the cytosol, leading to an environment that mimics Cys starvation, directing cells to an overall mechanism similar to oxidative- and copper stress responses [16]. Therefore, knowing the toxin substrate can help elucidate the specific TA pair functional impact, thus enriching the discussion when comparing bacteria, such as BCG strains, with known differences in the expression levels of the specific VapBC.

Another interesting VapBC feature was discovered for *Mtb* VapBC21. In this case, VapC21 can interact with its cognate VapB21, but also with the non-cognate VapB32 [45]. This shows that knowing the function of one TA pair may not be sufficient to understand its full impact, as different pairs may have overlapping interactions influencing the other’s functionality. From this point of view, differential VapBC47 expression due to RD16 loss in BCG Moreau may have a wider functional impact than what may be expected by only studying this pair. Further work unravelling the VapBC47 molecular mechanism will be very important to better understand the different BCG phenotypes.

Most studies about the VapBC family are focused on the structure and function of VapC toxins and their interaction with cognate VapB antitoxins [6,34,46,47,48]. Here, we show that *rv3407*-*rv3408* make up an operon and that its expression is increased in BCG Moreau because of the RD16 deletion. As previously shown, this deletion leads to *rv3406* constitutive expression in Moreau; here we corroborate that this mutation has a wider polar effect than previously detected, also interfering in the transcription of *rv3407* (*vapB47*) and *rv3408* (*vapC47*). Thus, in this strain the fine-tuned auto-regulation of the toxin–antitoxin expression by the antitoxin is likely bypassed, with unknown consequences to the physiology and immunomodulatory properties of this strain. We also show that rVapB47 binds DNA as predicted for this type of protein. Additionally, we identify the requirement of a palindrome in the *rv3407* 5´ region for rVapB47 DNA binding. These findings expand the current knowledge on the functional impact of the genomic differences between these important BCG vaccine strains and contribute to a better understanding of TA pairs functions.

## Data Availability

The data presented in this study are available in the article.
